# Effect of idiopathic thoracic scoliosis on the tracheobronchial tree

**DOI:** 10.1136/bmjresp-2017-000264

**Published:** 2018-03-25

**Authors:** James Farrell, Enrique Garrido

**Affiliations:** 1 School of Engineering, University of Edinburgh, Edinburgh, UK; 2 Scottish National Spine Deformity Service, Royal Hospital for Sick Children, Edinburgh, UK

**Keywords:** paediatric lung disaese, lung physiology, paediatric physician, thoracic surgery

## Abstract

**Introduction:**

High prevalence of obstructive lung disease has been reported in patients undergoing surgical correction of thoracic scoliosis. Airway narrowing due to spine morphology is analysed as a contributing factor.

**Methods:**

Preoperative surgical planning CTs of 34 patients with right-sided thoracic scoliosis (age: 17.6±9.0) were retrospectively analysed and compared with 15 non-scoliotic controls (age: 16.3±5.1). Three-dimensional models of spine and airway lumen were reconstructed. Based on thoracic sagittal profile, patients were divided into hypokyphosis (HypoS: <10°), normal kyphosis (NormS: ≥10° and <40°) and hyperkyphosis (HyperS: ≥40°) groups. Lumen area of bronchi, bifurcation angles and minimum spine–airway distance were measured. Pulmonary function tests were correlated to scoliosis, kyphosis and lumen area.

**Results:**

Loss of kyphosis led to proximity between bronchus intermedius (BI) and spine. HypoS (NormS) had lumen area reductions in the right main bronchus of 29% (19%), BI of 45% (23%), right middle lobar bronchus of 46% (32%) and right lower lobe bronchus (RLL7) of 66% (37%), respectively (P<0.05). The lower right superior segmental bronchus was reduced across all scoliotic groups (P<0.05). Airways were displaced caudal by 0.65±0.45 vertebra in patients with scoliosis. Loss of kyphosis correlated negatively with forced expiratory volume in 1 s/forced vital capacity (FEV_1_/FVC), FVC/(FVC predicted) and FEV_1_/(FEV_1_ predicted) (P<0.01). Lumen area of trachea, right upper lobar bronchus, BI and RLL7 correlated negatively with FEV_1_/FVC. BI and RLL7 narrowing were strong predictors of FVC and FEV_1_ loss (P<0.001).

**Conclusions:**

Right-sided main stem airways are narrowed in HypoS and NormS. Loss of kyphosis leads to narrowing of BI and its trifurcation. FEV_1_/FVC correlated negatively with airway narrowing, implying an obstructive element to lung function impairment in patients with scoliosis and hypokyphosis.

**Key messages**

- With decreasing kyphosis in right thoracic scoliosis, the spine intrudes into the thorax producing a rightward deflection of bronchus intermedius and its branches around the spine causing airway narrowing.
- Patients with increased airway resistance should be investigated for extrinsic bronchial compression by the scoliosis.
- Accurate diagnosis of the site of compression is essential to plan effective treatment to relieve the obstruction.

## Introduction

Scoliosis is a common disease in children, with an overall prevalence of 0.47%–5.2%.^1^ Thoracic scoliosis is a three-dimensional (3D) deformity involving the rib cage and spine. Eighty-two per cent of patients undergoing surgical correction of adolescent idiopathic scoliosis have the major curve in the thoracic spine.^2^ The main thoracic scoliosis is convex to the right, with the apical vertebra typically at T8 or T9.^3^ Left primary thoracic idiopathic curves are rare.^4^ In the axial plane the scoliosis rotates towards the right, frequently causing a loss of thoracic kyphosis in the sagittal plane.^5^ Seventy-five per cent of patients undergoing surgical correction have normal thoracic kyphosis, 14% have hypokyphosis and 11% have increased kyphosis.^2^

Long-term follow-up studies of patients with scoliosis have analysed the relationship of frontal spinal deformity and lung function. Patients with thoracic scoliosis exceeding 80° are likely to become symptomatic and develop dyspnoea, and very large scoliosis predisposes to cardiorespiratory failure.^6 7^ The restrictive effect of thoracic scoliosis on respiratory function is multifactorial due to a combination of decreased chest wall compliance, restricted rib movement, weakened respiratory muscle and reduced lung capacities.^8–11^ The location of the deformity, the degree of scoliosis and the loss of thoracic kyphosis correlate with loss of respiratory function.^11 12^

Although restrictive lung defects are the most prevalent pulmonary function abnormalities, obstructive or mixed lung disease with moderate to severe air trapping has been reported in up to 46% of patients undergoing preoperative evaluation for scoliosis surgery.^13^ McPhail *et al* 14 reported a prevalence of obstructive lung disease in 39% on patients scheduled for operative correction of thoracic scoliosis. Airway obstruction in patients with scoliosis and loss of thoracic kyphosis is probably more common than generally appreciated; early diagnosis allows specific planning of the scoliosis correction to restore lung function and to reduce postoperative complications.^15 16^

There is a paucity of information on the relationship of the thoracic deformity produced by the scoliosis and the effect on the bronchial tree. Extrinsic compression of the airway by the scoliosis causing loss of lung function may be wrongly attributed to progression of restrictive lung disease. The purpose of this study was to measure the 3D relationship of the spine and airways on CT reconstructions in patients with idiopathic right thoracic scoliosis. Subsequently, scoliosis, kyphosis and spine–airway proximity were correlated with airway narrowing and lung function to determine their relationships.

## Methods

### Subjects

After institutional review board approval, the surgeons’ imaging database (Kodak Carestream PACS) from 2008 to 2017 was searched retrospectively for preoperative planning CT scans of the thoracic spine of patients with idiopathic right thoracic scoliosis and apical vertebra between T7 and T10.^17^ A total of 34 preoperative scans met the inclusion criteria. Fifteen sex-matched and age-matched non-scoliotic subjects were obtained from normal oncology staging CT examinations as controls. Patients were matched for scoliosis Cobb angle and grouped based on CT Cobb angle measurements according to the Lenke criteria for hypokyphosis and hyperkyphosis of the thoracic spine.^18 19^ Non-scoliotic subjects were designated as the control group with coronal Cobb angles of less than 10° and a T5–T12 kyphosis Cobb angle between 10° and 40°. The subjects with scoliosis were classified into three groups: hypokyphotic scoliosis with T5–T12 kyphosis less than or equal to 10° (HypoS); scoliosis with normal sagittal profile with T5–T12 kyphosis between 10° and 40° (NormS); and kyphoscoliosis with T5–T12 kyphosis greater than 40° (HyperS). A summary of descriptive subject characteristics can be found in table 1. Patients underwent a multidetector CT (64-MDCT Siemens Somatom Scanner) at 0.6 mm collimation, 1 mm slice thickness and 0.7 mm reconstruction increments. Control CT scans were obtained as part of routine clinical care and scoliotic CT scans as part of preoperative planning.

**Table 1:** Subject group characteristics

Sagittal–coronal profile	Control	Hypokyphosis with scoliosis (HypoS)	Normal kyphosis with scoliosis (NormS)	Hyperkyphosis with scoliosis (HyperS)	Kruskal-Wallis test (P value)
Sample size, n	15	11	18	5	–
Age, year	16.3±5.1	20.2±7.8	17.1±10.4	13.2±5.3	0.119
Sex, n (female, %)	12 (80)	8 (72)	12 (66)	3 (60)	0.806
XR scoliosis Cobb	–	75.6±16.8	65.2±21.6	77.4±17.1	0.273
XR kyphosis Cobb T5–T12	–	3.3±13.3	30.3±12.5	60.8±6.5	<0.0001§¶**
CT scoliosis Cobb	4.1±2.1	64.1±15.9	59.3±19.4	77.2±17.4	<0.0001*†‡
CT kyphosis Cobb T5–T12	21.1±7.1	−4.6±12.1	19.8±6.5	51.0±9.2	<0.0001*‡§¶**
CT kyphosis Cobb T2–T12	32.1±7.5	3.4±15.6	28.1±10.3	52.7±8.9	<0.0005*‡§¶**
T-level count	–	6.4±0.9	6.5±1.8	5.8±1.3	0.590
Apical vertebra	–	9.0±1.1	8.3±1.1	8.0±1.4	0.311

Values are mean±SD.

Dunn’s post-hoc test: *P<0.05 for control vs HypoS, †P<0.05 for control vs NormS, ‡P<0.05 for control vs HyperS, §P<0.05 for HypoS vs NormS, ¶P<0.05 for HypoS vs HyperS, **P<0.05 for NormS vs HyperS.

XR, whole-spine standing radiographs.

### Structural variables

#### Image segmentation

CT scans were imported into Mimics V.18.0 (Materialise NV, Leuven, Belgium) in Digital Imaging and Communications in Medicine (DICOM) format. The spinal column from vertebrae T1 to T12 was isolated via Hounsfield thresholding and the masks of individual vertebrae manually segmented. The airways were segmented via a semiautomatic region growing method where missing airways or leakages into the parenchyma were manually edited. 3D models were created by wrapping and smoothing the masks in 3-matic V.10.0 (Materialise NV).

#### CT Cobb angle

**Figure 1:**
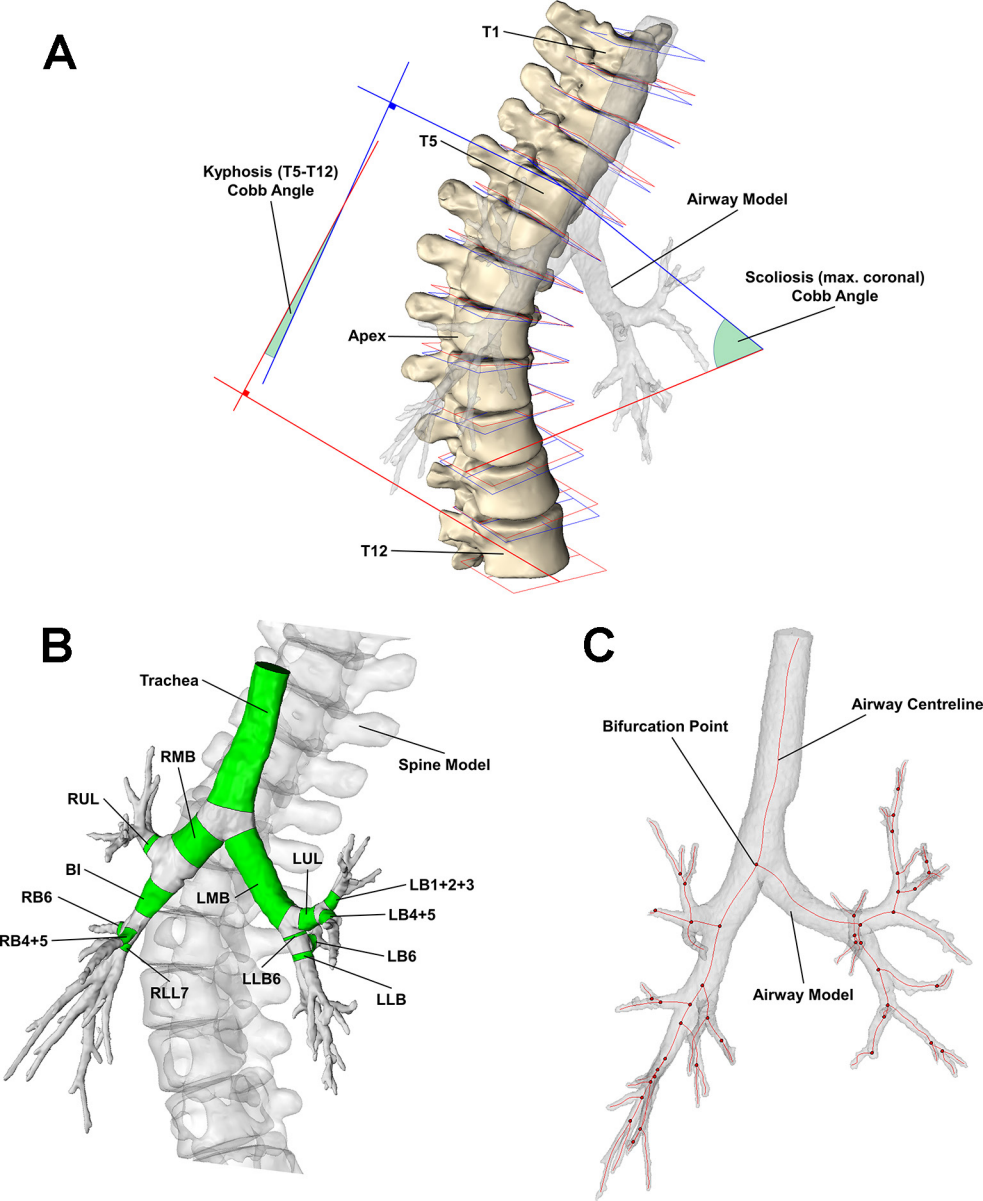
(A) Segmented spine model with best fit planes of the superior (blue) and inferior (red) endplates for each vertebra—endplate planes were used to define the scoliosis and kyphosis Cobb angles. (B) Airway model with individual airway segments (green). (C) Airway centreline (red lines) and bifurcation points (red points) of airway model. BI, bronchus intermedius; LB1+2+3, left apicoposterior and anterior segmental bronchus; LB4+5, left lingula bronchus; LB6, superior segment of the left lower lobe; LLB (LLB6), left lower lobe bronchus following (prior to) LB6 bifurcation; LMB, left main bronchus; LUL, left upper lobe bronchus; RB4+5, middle lobe bronchus; RB6, right lower lobe superior segmental bronchus; RLL7, right lower lobe bronchus; RMB, right main bronchus; RUL, right upper lobe bronchus.

A plane of best fit was constructed for the superior and inferior endplates of each vertebra (figure 1A) and the normal vector calculated; for a given pair of vertebrae, the coronal and sagittal Cobb angles were determined by calculating the angle between the superior and inferior vectors projected onto the coronal and sagittal planes, respectively. The scoliosis Cobb angle was defined as the maximum coronal Cobb angle and the kyphosis Cobb angle was measured between the T5 and T12 vertebrae.

#### Region of interest

The following airways were studied and shown in figure 1B: trachea, right main bronchus (RMB), right upper lobe bronchus (RUL), bronchus intermedius (BI), middle lobe bronchus (RB4+5), right lower lobe superior segmental bronchus (RB6), right lower lobe bronchus (RLL7), left main bronchus (LMB), left upper lobe bronchus (LUL), left apicoposterior and anterior segmental bronchus (LB1+2+3), left lingula bronchus (LB4+5), superior segment of the left lower lobe (LB6) and left lower lobe bronchus prior to and following LB6 bifurcation (LLB6 and LLB respectively).

#### Airway parameters

A skeleton centreline was generated from the 3D airway model. Airway segments were truncated along a surface perpendicular to the centreline, and the volume and length were documented for each airway segment. The average lumen area, A, was calculated by dividing the lumen volume by the segment length. Airway trajectories were calculated from proximal and distal bifurcation points of each airway (figure 1C). The bifurcation angles between daughter branches were determined via the rearrangement of the dot product from the vectors of the daughter branches. Airway trajectories were also measured anticlockwise from superior, anterior and superior reference trajectories in the axial, coronal and sagittal planes, respectively.

#### Spine–airway distance

The distance from the surface of the airway lumen to the spine was computed for the whole airway tree, d, and the minimum airway–spine distance, $\;d_{min}$, and the corresponding vertebra were documented for each airway segment.

#### Normalisation

Intersubject variability was accounted for by normalising airway lumen areas with the trachea lumen area measured at the T2 level, $A_{T2}$. The spine–airway distance was normalised by the T1–T12 spine length, $L_{T1-T12}$, which was determined by summing the distance between vertebrae centroids from T1 to T12. Normalised quantities are denoted with an asterisk (eg, $A^{*}$, $d^{*}$ for normalised lumen area and airway–spine distance, respectively). The mean $A_{T2}$ was 148±48 mm^2^ and 162±49 mm^2^; and the mean $L_{T1-T12}$ was 235±31 mm and 243±26 mm for controls and subjects with scoliosis, respectively.

### Lung function data

Spirometry (Jaeger MasterScreen PFT Pro) via forced manoeuvres was performed for subjects with scoliosis in accordance with the joint American Thoracic Society and European Respiratory Society standards.^20^ Forced expiratory volume in 1 s (FEV_1_) and forced vital capacity (FVC) were recorded with the predicted values (FEV_1pred_ and FVC_pred_, respectively) in accordance with validated reference data.^21^ Arm span was measured and taken to be representative for height in correcting for ageing and height gain when calculating lung function predicted values.^22 23^

### Statistics

The non-parametric one-way analysis of variance by ranks (Kruskal-Wallis) test followed by post-hoc pairwise multiple comparisons Dunn test were performed to determine whether airway characteristics differed between groups. In addition, Pearson correlations between airway, spinal and lung function variables were performed. A significance level of 0.05 was used for all tests. Multivariate regression of subjects with scoliosis was conducted on lung function using kyphosis and scoliosis Cobb angles as predictors. All statistical analyses were generated using the R software (http://www.R-project.org).

## Results

CT-measured scoliosis and kyphosis gave Pearson correlation coefficients of 0.98 and 0.85, respectively, when compared with their radiographic standing equivalents (P<0.001). Scoliosis was reduced by 11.5°, 5.9° and 0.2° from standing to supine, while kyphosis decreased by 7.9°, 10.5° and 9.8° for HypoS, NormS and HyperS groups, respectively.

### Airway–spine relationship

**Figure 2:**
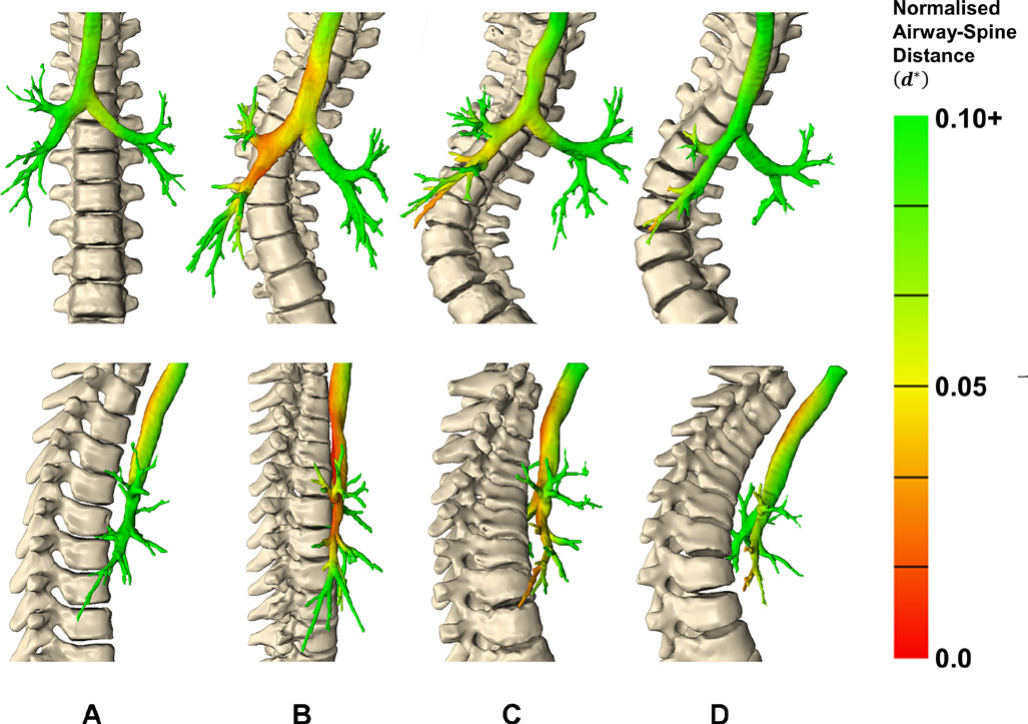
Normalised airway–spine minimum distances for representative subjects from (A) control, (B) hypokyphosis, (C) normal kyphosis and (d) hyperkyphosis. The top row displays the coronal view anterior to the subject and the bottom row displays the sagittal views right of the subject.

Figure 2 shows $d^{*}$ throughout the airway tree for representative subjects from each group. Subjects with HypoS and NormS showed reduced $d_{min}^{*}$ for RMB, RUL, BI, RB4+5, RB6 and RLL7 when compared with controls (P<0.05). Reduced spine-airway proximity occurs in the region of the BI and its bifurcation, which is more pronounced in the subject with HypoS. A mean reduction of 87%, 62% and 4% in $d_{min}^{*}$ for BI was observed for HypoS, NormS and HyperS, respectively. When comparing the airway and spine landmarks, it was found that airway bifurcation points in patients with scoliosis were 0.65±0.45 vertebral bodies lower than in controls. In patients with scoliosis, the bifurcation of RMB, BI and RLL was at the level of vertebral bodies T6, T7 and T7/8 discs, respectively. $A^{*}$ was positively correlated with $d_{min}^{*}$ for RMB, BI, RB4+5, RB6 and RLL7 (r=0.46, 0.62, 0.51, 0.64 and 0.65, respectively; P<0.05).

### Lumen area

Normalised lumen areas as a fraction of the control group mean, $A^{*}/\overset{-}{A_{Control}^{*}}$, are displayed in table 2. The majority of differences appeared in right-sided airways with high reductions in airways surrounding the BI trifurcation, while left-sided airways were not significantly affected other than LMB in HypoS. $A_{BI}^{*}$ was reduced by 45% and 23% in subjects with HypoS and NormS, respectively, while subjects with HyperS did not appear to be significantly affected. Significant reductions in RMB, RB4+5 and RLL7 were observed when comparing HypoS and NormS with controls. RB6 was reduced by approximately 40%–60% across all scoliotic groups.

**Table 2:** Normalised lumen area as a fraction of control group mean $\left(A^{*}/\overset{-}{A_{control}^{*}}\right)\;$ among subjects with HypoS, NormS and HyperS

Airway segment	Hypokyphosis with scoliosis (HypoS)	Normal kyphosis with scoliosis (NormS)	Hyperkyphosis with scoliosis (HyperS)	Kruskal-Wallis test (P value)
Trachea	0.89±0.06	0.95±0.10	0.95±0.08	<0.005*§
RMB	0.71±0.15	0.81±0.13	0.85±0.18	<0.0005*†
RUL	0.62±0.32	0.85±0.33	0.75±0.24	<0.05
BI	0.55±0.22	0.77±0.17	0.92±0.21	<0.0001*†¶
RB4+5	0.54±0.28	0.68±0.26	0.93±0.27	<0.005*†
RB6	0.38±0.37	0.57±0.40	0.45±0.31	<0.005*†‡
RLL7	0.34±0.32	0.63±0.31	0.86±0.29	<0.0005*†
LMB	0.74±0.20	0.88±0.13	0.86±0.27	<0.05*
LUL	0.88±0.12	0.98±0.32	1.02±0.38	0.420
LB1+2+3	0.89±0.36	0.85±0.48	0.62±0.37	0.079
LB4+5	0.78±0.26	0.83±0.38	1.30±0.66	0.116
LLB6	0.87±0.20	0.84±0.23	0.93±0.14	0.255
LB6	0.86±0.30	0.76±0.38	0.58±0.04	0.072
LLB	0.82±0.27	0.81±0.31	0.84±0.13	0.131

Dunn’s post - hoc test: *P< 0.05 for c ontrol vs HypoS, † P<0.05 for control vs NormS, ‡P<0.05 for control vs HyperS, §P<0.05 for HypoS vs NormS, ¶ P<0.05 for HypoS vs HyperS.

BI, bronchus intermedius; LB1+2+3, left apicoposterior and anterior segmental bronchus; LB4+5, left lingula bronchus; LB6, superior segment of the left lower lobe; LLB (LLB6), left lower lobe bronchus following (prior to) LB6 bifurcation; LMB, left main bronchus; LUL, left upper lobe bronchus; RB4+5, middle lobe bronchus; RB6, right lower lobe superior segmental bronchus; RLL7, right lower lobe bronchus; RMB, right main bronchus; RUL, right upper lobe bronchus.

### Bifurcation angles and airway trajectories

Bifurcation angles were found to be significantly reduced between the daughter branches of BI (namely RB4+5, RB6 and RLL7) for subjects with HypoS and NormS. Narrowing of BI, RB6 and RLL7 correlated with the narrowing of trifurcation angles of the BI (r=0.55–0.68, P<0.0001). Differences in airway trajectories were present with RB6 being rotated towards the right in the axial plane by 22°±13° in subjects with scoliosis (P<0.05). In the coronal plane, significant differences were measured in BI (P<0.001) and RLL7 (P<0.001) between groups. BI was more horizontal in the coronal plane by 14°, 10° and 8° in HypoS, NormS and HyperS, respectively, from a control average of 156°±4°. As BI, RLL7 was elevated by a mean of 16.6°±4° in subjects with scoliosis when compared with the control mean of 148±8°. In the sagittal plane, RB4+5 was angled inferiorly by 27°, 10° and 9° from the control mean of 119°±6° in HypoS, NormS and HyperS, respectively (P<0.001).

### Structural and lung function correlations

**Figure 3:**
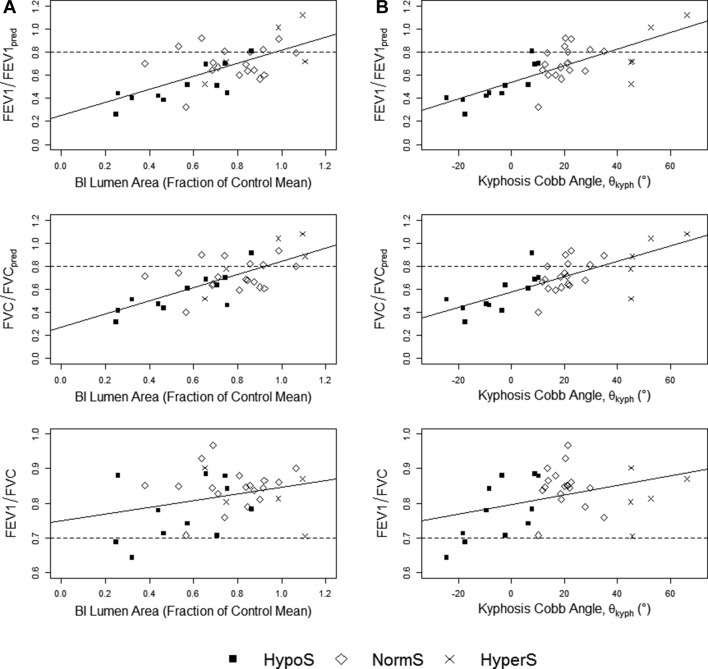
Plots of FEV_1_/FEV_1pred_, FVC/FVC_pred_ and FEV_1_/FVC against (A) BI lumen area and (B) CT-measured kyphosis angle with least squares regression lines. HypoS—hypokyphosis with scoliosis (black square); NormS—scoliosis with normal sagittal profile (white diamond); HyperS—kyphoscoliosis (cross). BI, bronchus intermedius; FEV_1_, forced expiratory volume in 1 s; FEV_1pred_, FEV_1_ predicted; FVC, forced vital capacity; FVC_pred_, FVC predicted.

Pearson correlations were calculated between lung function and $A^{*}$ and for each airway to assess the impact of airway narrowing on lung function. FVC/FVC_pred_ correlated strongly with $A^{*}$ for BI and RLL7 (r=0.77 and 0.61, respectively; P<0.001) showing significant correlations with RUL, RB4+5 and RB6 (r=0.44, 0.49 and 0.4; P<0.05). FEV_1_/FEV_1pred_ correlated strongly with $A^{*}$ for BI and RLL7 (r=0.66 and 0.55, respectively; P<0.001) showing significant correlations with RUL, RB4+5 and RB6 (r=0.34, 0.41 and 0.34; P<0.05). FEV_1_/FVC correlated with $A^{*}$ of the trachea, RUL, BI and RLL7 (r=0.46, 0.43, 0.48 and 0.42, respectively; P<0.05). Overall, there was a positive correlation between lung function and the lumen area of right-sided, main stem airways. Figure 3A shows the relationship between the lung function ratios and BI lumen area.

For patients with scoliosis, multivariable linear regressions were performed to determine the contribution of scoliosis and kyphosis to FVC/FVC_pred_, FEV_1_/FEV_1pred_ and FEV_1_/FVC, respectively. CT-measured Cobb angles were used and are measured in degrees. Subjects’ FVC/FVC_pred_ is equal to 0.60242–0.00047 (scoliosis) +0.00605 (kyphosis) (*F*(2,34)=8.307, P<0.001, $R_{adj}^{2}$ of 0.369); FEV_1_/FEV_1pred_ is predicted by 0.56333–0.00038 (scoliosis) +0.00724 (kyphosis) (*F*(2,34)=7.497, P<0.001, $R_{adj}^{2}$ of 0.314); and FEV_1_/FVC was equal to 0.76842+0.00037 (scoliosis) +0.00225 (kyphosis) (*F*(2,34)=5.692, P<0.005, $R_{adj}^{2}$ of 0.2811). Kyphosis was significant in all models (P<0.05) with subjects losing 0.61%, 0.72% and 0.22% in FVC/FVC_pred_, FEV_1_/FEV_1pred_ and FEV_1_/FVC, respectively, for each degree in kyphosis lost. Scoliosis was found not to be a significant predictor in any of the models due to study design as subjects were matched for scoliosis. The relationship between kyphosis and lung function is shown in figure 3B.

## Discussion

Our results demonstrate that close proximity between spine and airway results in significant airway narrowing. Distal to the RMB, loss of kyphosis was a strong predictor of airway stenosis. The BI was reduced by 45% and 23% in HypoS and NormS, respectively, but not significantly affected in HyperS. Patients with HypoS had on average a 42%, 62% and 66% reduction of $A^{*}$ in RB4+5, RB6 and RLL7, respectively. Interestingly, $A_{RB6}^{*}$ was significantly narrowed in all scoliotic groups when compared with controls; this is likely to be due to the posterior trajectory of RB6, which places it in close proximity to the laterally displaced spine. Previous reports have shown that bronchial compression occurs on the convex side of a right thoracic scoliosis, causing ventilation defects in the middle and/or right lower lobe. Slit-like anteroposterior (AP) compression of the BI has been described on bronchoscopy.^15 24 25^

**Figure 4:**
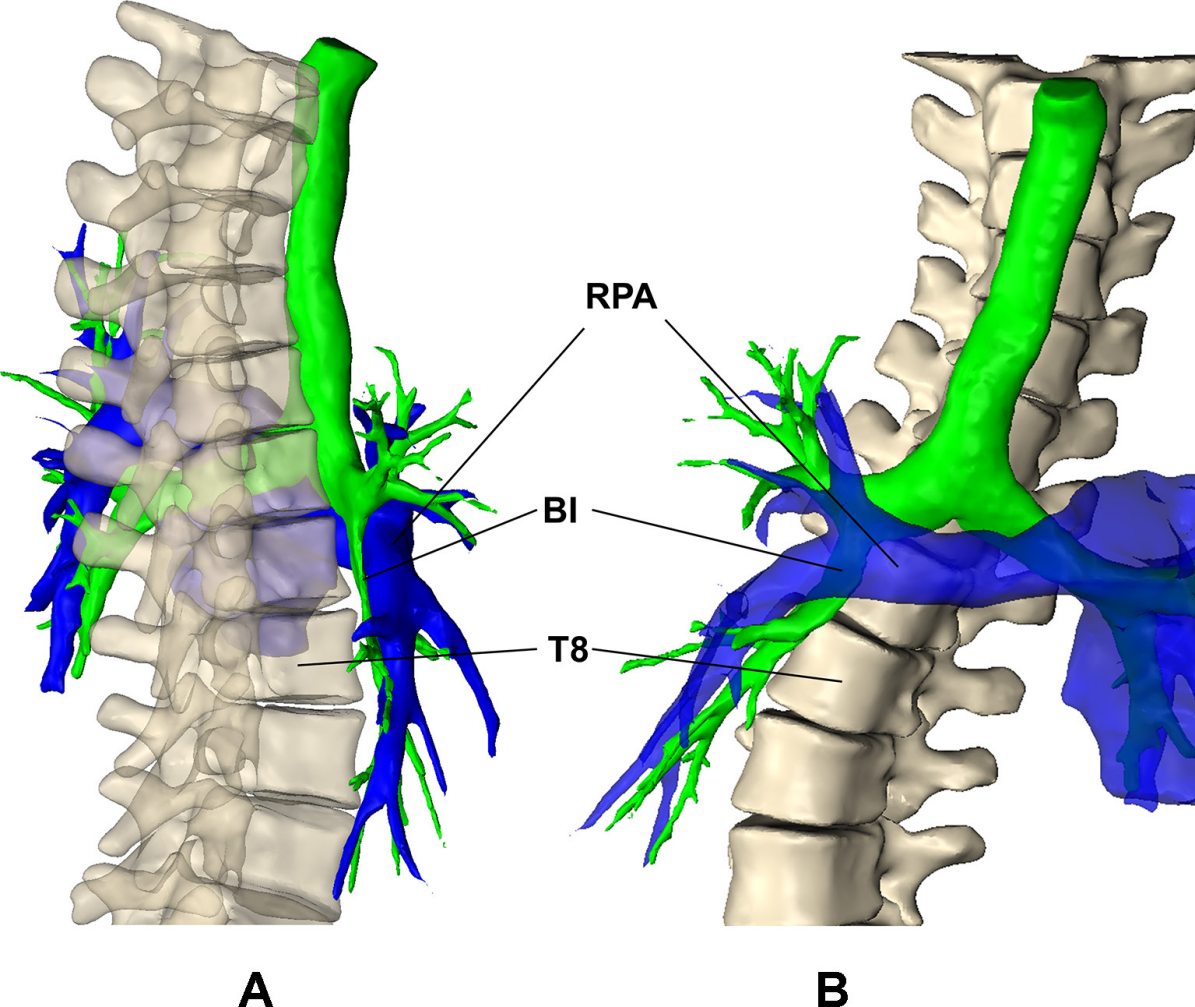
Subject with hypokyphosis with T1–T10 (bone), airway lumen (green) and pulmonary artery (blue) displayed from the (A) sagittal and (C) coronal perspectives. BI, bronchus intermedius; RPA, right pulmonary artery.

The trajectory of the BI and RLL7 showed a more horizontal trajectory with decreasing kyphosis. We hypothesise that the anterior protrusion of the spine produces a rightward deflection of the trajectory of BI and RLL7. Furthermore, the right hemithorax is rotated posteriorly wrapping the airway around the spine. Anteriorly, the right pulmonary artery or interlobar artery crosses anterior to the BI, and it seems plausible that the vessel has a causative role in the airway impingement (figure 4).^26^

Winter *et al* 27 reported decreased vital capacities due to reduced AP diameters of the chest in patients with HypoS. Our results demonstrated that hypokyphosis correlated negatively with FVC/FVC_pred_, FEV_1_/FEV_1pred_ and FEV_1_/FVC. In 1970, Bjure *et al* already indicated that airway closure was an aetiological factor of lung function impairment in patients with severe scoliosis.^28^ Dubousset *et al* reported direct airway compression by the scoliosis. They described the Spinal Penetration Index (SPI), which is a transverse plane measurement obtained by CT to quantify the spinal intrusion into the thorax.^29^ High SPI measured with biplanar stereoradiographic reconstruction has shown to correlate with the presence of obstructive lung disease.^30^ It is likely that different pathophysiological mechanisms result in the loss of lung function in patients with scoliosis. Disturbed chest mechanics and reduced lung volumes lead to a restrictive lung defect, while airway narrowing increases airway resistance, particularly in patients with severe scoliosis and decreased thoracic kyphosis, causing a mixed ventilatory defect. Based on the results, spirometric evaluation of patients with scoliosis and reduced thoracic kyphosis is recommended. Patients with reduced FEV_1_/FVC ratios or FVC <65% predicted are likely to suffer significant pulmonary morbidity and should undergo further diagnostic workup.^11^ Flow volume patterns are frequently normal in patients with bronchomalacia.^25 31^ Air trapping reflected in an increased ratio of residual lung volume to total lung capacity and increased airway resistance can indicate airway narrowing with expanded pulmonary function testing by body plethysmography.^23 32^

Bronchial obstruction leads to physiological disturbance based on location, degree of narrowing and the history of the stenosis. Our morphological analysis has shown that with increased hypokyphosis, airway narrowing begins more proximal at the BI, affecting airflow into the right middle and lower lobes which contribute 9% and 25% respectively to total lung volume.^31^ Several mechanisms can exacerbate the stenosis of the airway. Increased intrapleural pressure during expiration narrows the airway, increasing resistance and decreasing flow. Bernoulli’s principle states that increased velocity through the narrowed airway occurs simultaneously with a decrease in airway pressure. The more stenotic the airway and the more forceful the expiration, the more likely the airway will obstruct.^33^ Early recognition of airway stenosis is important before obstruction becomes chronic, causing atelectasis and recurrent infection with irreversible loss of lung function.^16 34^

Location and characterisation of the narrowing are paramount to plan surgical correction of the scoliosis and decompress the obstructed airway. There is no agreement on the diagnostic workup for extrinsic compression of the airway by scoliosis. Bronchoscopy with forced expiratory manoeuvres is the current ‘gold standard’ for the diagnosis of bronchomalacia of the trachea and main stem bronchi.^33^ Bronchoscopy is performed under sedation and requires the patient to inhale and forcibly exhale when instructed. Due to the invasive nature and risk of complications, it may not be appropriate to perform bronchoscopic examinations in the immediate period prior to scoliosis correction, particularly in patients with impaired respiratory function.

CT scanning allows objective delineation of the location, extent and adjacent relationships of anatomical structures, including spine and vasculature causing extrinsic airway compression. CT allows simultaneous evaluation of lung and spine for surgical planning of the scoliosis correction.^35^ Low-dose dynamic CT including end-inspiratory and dynamic-expiratory imaging has shown a high level of concordance with bronchoscopy in the diagnosis of tracheobronchomalacia.^36^ There is potential for high radiation doses with CT; thus, adherence to paediatric guidelines to produce diagnostic images without excessive radiation exposure is mandatory.^37^ Dynamic volumetric CT technique has demonstrated the ability to obtain diagnostic images at low radiation dose and much less than previous paired inspiratory and expiratory CT techniques.^38^

Ventilation/perfusion scanning is not used routinely in the assessment of patients with scoliosis but can provide a regional functional assessment in patients with significant respiratory symptoms. Krypton-81m as a ventilation agent with its ability to assess tidal breathing has been used to assess the posture-dependent right bronchial obstruction in patients with scoliosis.^39^

In the future, hyperpolarised helium-3 MRI may provide accurate airway lumen measurement and dynamic imaging with regional lung function assessment without the use of ionising radiation. It is likely that these techniques will lead to a better understanding of the pathophysiology of respiratory disease in scoliosis, translating to improved care and specific scoliosis correction techniques.

### Study limitations

There are several weaknesses to this study. The CT scans analysed in this study were performed as preoperative planning scans prior to correction of the scoliosis via spinal instrumentation and were not controlled with respect to respiratory phase. Thus, end-expiration lumen reductions were not captured. Second, the scans were performed on larger deformities with abnormal sagittal profiles and as such represent a more severe sample of patients with idiopathic scoliosis. Idiopathic hyperkyphosis is rare and consequently our sample size is relatively small. Third, the scans were obtained in the supine position, changing the coronal and sagittal alignment of the spine. Posture-dependent lung function changes have been demonstrated in patients with scoliosis. It is possible that the supine position further decreases thoracic kyphosis, producing additional narrowing of the airways.^39 40^

## Conclusion

The preoperative morphological analysis of large airways in patients with right idiopathic thoracic scoliosis demonstrated that the loss of thoracic kyphosis causes right-sided airway narrowing. More severe hypokyphosis led to more proximal and severe narrowing in the BI. FEV_1_/FVC correlated negatively with airway narrowing, implying an obstructive element to lung function impairment in patients with scoliosis and loss of kyphosis. Preoperative diagnosis of extrinsic airway narrowing by the scoliosis is essential to plan scoliosis correction techniques that can adequately decompress the airway. Although decrease in lung function in patients with scoliosis is multifactorial, morphological changes in airways from variance in the sagittal profile play an more important role in impairing lung function than is generally appreciated.
